# Evidence-Based Mechanical Ventilatory Strategies in ARDS

**DOI:** 10.3390/jcm11020319

**Published:** 2022-01-10

**Authors:** Adnan Liaqat, Matthew Mason, Brian J. Foster, Sagar Kulkarni, Aisha Barlas, Awais M. Farooq, Pooja Patak, Hamza Liaqat, Rafaela G. Basso, Mohammed S. Zaman, Dhaval Pau

**Affiliations:** 1 Southeast Health, Dothan, AL 36301, USA; aliaqat@southeasthealth.org (A.L.); bjfoster@southeasthealth.org (B.J.F.); skulkarni@southeasthealth.org (S.K.); amfarooq@southeasthealth.org (A.M.F.); papatak@southeasthealth.org (P.P.); rgiuntibasso@southeasthealth.org (R.G.B.); mszaman@southeasthealth.org (M.S.Z.); 2 Alabama College of Osteopathic Medicine, Dothan, AL 36303, USA; masonm@acom.edu; 3 Mercy Health, Rockford, IL 61114, USA; aishab_93@hotmail.com; 4 Wah Medical College, Wah Cantt 47040, Pakistan; 98hamzaliaqat@gmail.com

**Keywords:** acute respiratory distress syndrome, ARDS, mechanical ventilation strategies, PEEP, lung recruitment, noninvasive ventilation, ECMO

## Abstract

Acute respiratory distress syndrome (ARDS) remains one of the leading causes of morbidity and mortality in critically ill patients despite advancements in the field. Mechanical ventilatory strategies are a vital component of ARDS management to prevent secondary lung injury and improve patient outcomes. Multiple strategies including utilization of low tidal volumes, targeting low plateau pressures to minimize barotrauma, using low FiO_2_ (fraction of inspired oxygen) to prevent injury related to oxygen free radicals, optimization of positive end expiratory pressure (PEEP) to maintain or improve lung recruitment, and utilization of prone ventilation have been shown to decrease morbidity and mortality. The role of other mechanical ventilatory strategies like non-invasive ventilation, recruitment maneuvers, esophageal pressure monitoring, determination of optimal PEEP, and appropriate patient selection for extracorporeal support is not clear. In this article, we review evidence-based mechanical ventilatory strategies and ventilatory adjuncts for ARDS.

## 1. Introduction

**Figure 1:**
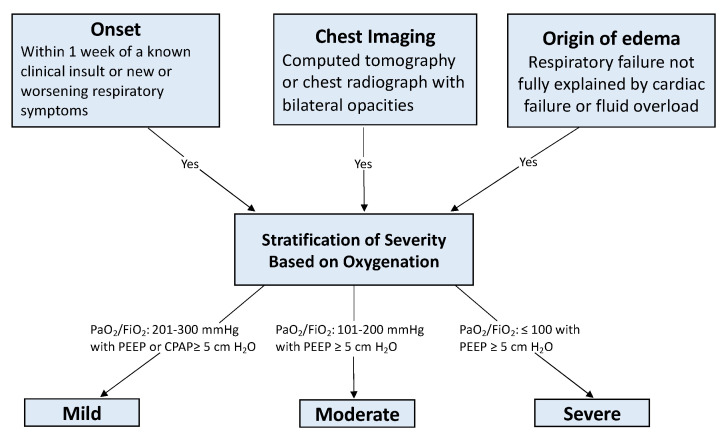
Berlin Diagnostic Criteria [2]. Abbreviations: PaO_2_ = arterial partial pressure of oxygen; FiO_2_ = fraction of inspired oxygen; PEEP = positive end expiratory pressure; CPAP = continuous positive airway pressure.

Acute respiratory distress syndrome (ARDS) is an acute, severe lung injury that is characterized by inflammatory cascades, hypoxemia, and diffuse lung involvement. In 1967, ARDS was first described as an acute hypoxic lung injury with infectious and traumatic triggers, similar to neonatal congestive atelectasis and hyaline membrane disease [1]. Since then, several definitions of ARDS have been established, but the Berlin definition is the latest and most widely accepted [2]. The diagnostic criteria are summarized as an acute injurious lung event with diffuse bilateral lung opacities of non-cardiogenic origin on imaging (See Figure 1).

ARDS, in regards to incidence, morbidity, and mortality, is a sinister clinical conundrum—a condition that is both common and devastating. The age-adjusted incidence of ARDS in individuals with PaO_2_/FiO_2_ (arterial partial pressure of oxygen/fraction of inspired oxygen) ratio ≤ 300 mmHg is 86 per 100,000 person-years and 64 per 100,000 person-years for individuals with PaO_2_/FiO_2_ ratio ≤ 200 mmHg [3]. This approximates to 10% of intensive care unit (ICU) patients and 23% of patients on mechanical ventilation [4]. Patients between the ages of 15 and 19 have a 24% mortality, whereas older patients suffer to a greater degree with mortality rates approaching 60% [3].

**Figure 2:**
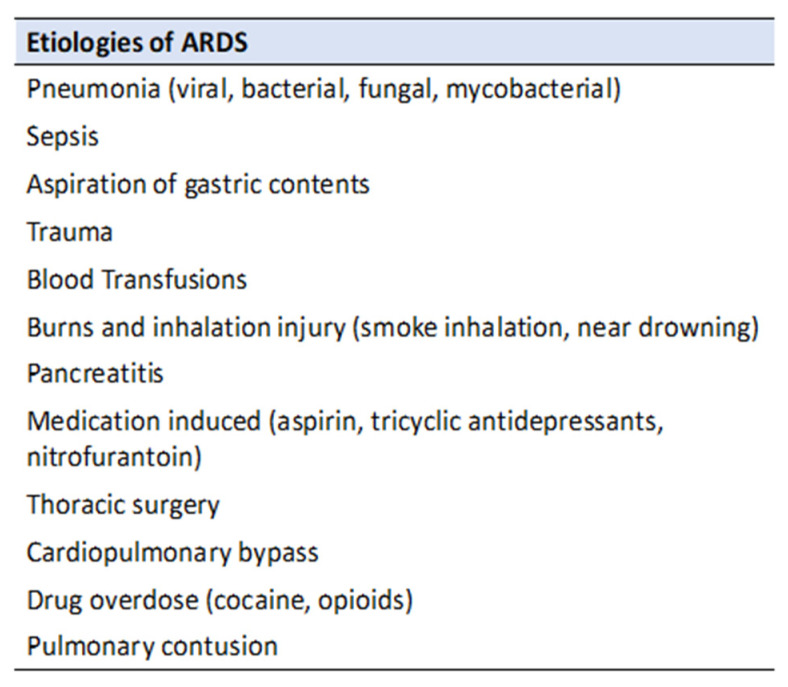
Common etiologies of ARDS.

There are many well established and potential etiologies of ARDS (see Figure 2), but around 85–90% are caused by pneumonia, sepsis, aspiration of gastric contents, trauma, or blood transfusion [5,6,7]. The hallmark pathophysiological mechanisms of ARDS follow a predictable pattern that begins with an initial insult with a subsequent inflammatory response, which leads to endothelial damage and increased pulmonary capillary permeability. The subsequent proliferation and fibrosis stages attempt to repair the initial inflammatory insult and endothelial damage. More specifically, the fibrotic phase recruits fibroblasts and implements repair mechanisms that cause intra-alveolar fibrosis and capillary obliteration [6,7]. This intra-alveolar architectural change leads to prolonged mechanical ventilation and increased mortality [6].

When ARDS was first described in 1967, Ashbaugh et al. identified that positive end expiratory pressure (PEEP) was the most efficacious intervention to improve outcomes [1]. Since then, more advanced treatment modalities have been developed to improve patient outcomes, albeit with varying degrees of success. In this article, we review current evidence-based ventilatory strategies and ventilatory adjuncts, as well as their mechanistic role in the management of patients with ARDS.

## 2. Lung Protective Ventilation

Lung protective ventilation is the mainstay of ventilatory management of patients with ARDS and plays a critical role in improving clinical outcomes. Lung protective ventilation strives to prevent over-distention, or “stretch”, of the aerated lung, as this has been shown to disrupt both the pulmonary endothelium and epithelium, resulting in lung inflammation, atelectasis, hypoxemia, and the release of inflammatory mediators [8]. It is recommended that patients be ventilated with low tidal volumes (4–8 mL/kg of predicted body weight) [8]. Many of these parameters were derived from the ARMA trial conducted by ARDSNet investigators [8]. This landmark study demonstrated significantly reduced hospital mortality and duration of mechanical ventilation when a lung protective ventilatory strategy was utilized [8]. Prior to this study, traditional ventilation strategies focused on increasing arterial oxygen saturation with the use of higher tidal volumes at the expense of alveolar distension. This contributed to stretch induced disruption of the alveolar endothelium and a potentiation of the innate inflammatory response—further exacerbating the underlying mechanism of ARDS. The ARMA protocol derived from the ARDSNet trial provided evidence that reducing alveolar stretch injury with low tidal volumes (6 mL/kg of predicted body weight) improves survival and was subsequently adopted as the mainstay of ARDS ventilatory management [8].

Lung protective ventilation includes the use of high PEEP but this has a less defined benefit in ARDS management. In the ARMA trial, PEEP was set according to the amount of FiO_2_ a patient needed, using a titration table. Studies such as the ALVEOLI trial reveal that utilization of an even higher PEEP titration table does not result in improvement in patient outcomes [9]. Thus, though the utilization of high PEEP is a part of lung protective ventilation, “how high” is not clearly defined.

In addition, lung protective ventilation includes targeting low plateau pressures [10]. Plateau pressures can be referred to as a measure of pulmonary compliance. It is generally accepted that plateau pressures are tolerable up to 30–32 cm H_2_O. The ARMA trial also found that coupled with low tidal volume, a lower plateau pressure helps to reduce mortality in patients with ARDS [8]. Plateau pressures in turn can help risk stratify cut-off values during mechanical ventilation for potential barotrauma and lung parenchyma injury. A better understanding of mechanical ventilatory support and its related ventilator induced lung injury (VILI) is an essential component of managing patients with ARDS. Failure to comply with lung protective ventilation strategies might enhance the risk of VILI which involves excessive stress to the lungs (high transpulmonary pressure), and increased strain (alveolar-overdistention) which eventually leads to barotrauma, alveolar rupture, and development of pulmonary edema [11,12].

## 3. Optimal PEEP

Adjustment of PEEP is an important strategy to improve oxygenation in patients with ARDS. Although no single PEEP strategy has proven to be ideal, several well-known trials have outlined various PEEP titration strategies with the underlying consensus surrounding the idea of limiting over-distention of lung parenchyma. In the ARMA trial, PEEP and FiO_2_ were titrated to target an oxygen saturation of 88 to 95 percent or PaO_2_ of 55 to 80 mmHg. Although PEEP levels ranging from 5 to 24 cm H_2_O were used, the trial outlined the need for dynamic changes to PEEP in combination with FiO_2_. In addition to achieving target O_2_ saturation and PaO_2_ levels, this PEEP strategy also involves ensuring low plateau pressures, a fundamental part of classical lung protective ventilation [8].

Following the ARMA trial, the EXPRESS trial further delineated the recommendation for lung protective ventilation with a high PEEP, low-tidal volume strategy. The EXPRESS trial revealed that a high PEEP strategy (up to 14.6 cm H_2_O) was preferable to a moderate PEEP strategy (5 to 9 cm H_2_O), again with specific titration of PEEP to target plateau pressures of less than 30 cm H_2_O [13]. It is important to note that the EXPRESS trial did not show significantly reduced mortality rates with the use of higher PEEP, but instead associated high PEEP with improved lung function and reduced duration of mechanical ventilation [13].

The LOVS trial investigated an open lung approach, described as a combination of low tidal volume, high PEEP, and lung recruitment maneuvers. A mean PEEP of 14.6 cm H_2_O (versus 9.8 cm H_2_O in the control group) was used. As noted in previous trials, special considerations were taken to ensure that plateau pressures were kept low to maintain lung protective measures; no greater than 40 cm H_2_O. The LOVS trial showed no difference in all-cause hospital mortality or amount of barotrauma when comparing a high PEEP strategy with an established low-tidal volume standardized ventilation strategy [14].

Although previous trials did not find significantly reduced mortality rates with use of high PEEP versus moderate or low PEEP, it must be mentioned that one meta-analysis did demonstrate mortality benefit with the use of high PEEP versus lower levels of PEEP in a specific subset of patients found to have baseline PaO_2_/FiO_2_ ratios below 200 mmHg [15].

Finally, various techniques for adjusting PEEP based on alternative intrinsic pressures have recently been defined, paving the way for future research. Notably, the EPVENT trial investigated the use of esophageal balloon catheters to measure esophageal pressure (an estimate of transpulmonary pressure) as a basis for adjustment of PEEP [14]. This technique was compared to that used in the ARMA trial and found to be superior in improving oxygenation [16]. However, a subsequent trial, EPVENT2, revealed that in patients with moderate-to-severe ARDS, titration of PEEP guided by esophageal pressure did not significantly improve mortality or reduce days free from mechanical ventilation, although it did result in use of less rescue therapy, notably need for extracorporeal membrane oxygenation (ECMO) [17]. A reanalysis of the EPVENT-2 trial was subsequently conducted to further evaluate the effect of esophageal pressure guided PEEP and empirical use of high PEEP on survival. This trial revealed that the PEEP titration closer to 0 cm H_2_O was associated with better survival, irrespective of severity of multi-organ dysfunction [18].

Given the complexity of factors involved in titration of PEEP, and the various influences on this parameter, no single PEEP strategy has been outlined as ideal by current literature. The consensus still remains that dynamic adjustment of PEEP to ensure low plateau pressures, aligning with an overall lung protective strategy, is more important than the level of PEEP itself.

## 4. Driving Pressure

Driving pressure is defined as the ratio between tidal volume and pulmonary compliance. It can be estimated by calculating the difference between plateau pressure and PEEP [19]. A multilevel mediation analysis involving 3562 individual patients from nine previously performed randomized trials revealed that ventilator settings leading to a decrease in driving pressure were associated with increased survival in patients with ARDS [20]. Driving pressures below 14 cm H_2_O are associated with better outcomes [4]. Several methods, including reduction of tidal volume, optimization of PEEP, and recruitment maneuvers can improve lung compliance and reduce driving pressure [21]. Additionally, neuromuscular blockers, prone positioning, and extracorporeal measures can be utilized as adjuncts to reduce driving pressure [22]. Recently, Costa and colleagues suggested a model of “4 × ΔP + respiratory rate” as a surrogate for mechanical power, which is associated with increased mortality [23]. This implies that in terms of lung damage, a 1 point reduction in driving pressure is equivalent to 4 point reduction in respiratory rate [23]. Since driving pressure represents the tidal volume adjusted according to the compliance of the respiratory system, it may lead to a reduction in lung stretch, thereby reducing mortality [24]. Adjusting ventilator parameters including tidal volume based upon driving pressure rather than predicted body weight may result in improved outcomes. However, there is a lack of evidence for a specific ventilation strategy to achieve a lower driving pressure. Targeting a driving pressure below 14 cm H_2_O is reasonable, but additional evidence is needed to prove that ventilation strategies targeting low driving pressures can improve the outcomes of patients with ARDS.

## 5. Recruitment Maneuvers

Recruitment maneuvers involve increasing airway pressures for a short period of time with the aim to expand collapsed alveoli, allowing for better gas exchange. Recruitment maneuvers help enhance lung compliance and improve gas exchange, but their impact on patient outcomes is not well defined in the literature. The recruitment action should last at least 7 to 8 seconds [25]. One method involves increasing tidal volume gradually, using a low respiratory rate in volume-controlled ventilation, or by gradual up-titration of PEEP while maintaining driving pressure until a peak inspiratory pressure of at least 40 cm H_2_O is reached in pressure control ventilation [26,27]. One large randomized controlled trial has shown that titration of PEEP to respiratory compliance with recruitment maneuvers; compared to maintaining low PEEP is associated with increased mortality [9,28]. In another trial, lung recruitment failed to show any mortality benefit or reduce ventilator free days, but limited the use of adjunctive therapies [29]. Recently, a newer bedside technique, recruitment to inflation ratio (R/I) has been proposed on the basis of balance between recruitment and overinflation [30]. R/I ratio was found to be associated with better oxygenation [30]. In one recent study, R/I ratio was used to assess lung recruitability, particularly in COIVD-19 related ARDS, but its use can be considered in non-COVID-19 related ARDS [30]. Recruitment maneuvers can provide a temporary benefit by improving compliance and gas exchange at the expense of increased sedation requirements, decreased cardiac preload, increased distension of alveoli causing barotrauma, and possibly worsening oxygenation by promoting perfusion of poorly ventilated areas.

## 6. Prone Ventilation

Prone positioning has been a widely accepted and well-utilized tool for the management of ARDS. Various randomized, controlled trials have confirmed that oxygenation is significantly improved when patients are in the prone position compared to the supine position [31,32]. In addition, a meta-analysis showed that prone ventilation can improve mortality of patients with severe ARDS [33]. Improved survival is not mediated through improved oxygenation but rather due to a more even distribution of volume and distention forces across the lung, leading to a reduction of ventilator-induced lung injury [33]. Most recent guidelines recommend daily sessions of prone ventilation in patients suffering from severe ARDS (See Table 1) [6]. The PROSEVA trial showed that early application of prolonged proning sessions was found to significantly decrease 28-day and 90-day mortality [29]. Patients in the PROSEVA trial underwent a mean prone session duration of 17 ± 3 h [34]. Other clinical trials have confirmed this number, citing optimal proning time to be at least 16 h per day in patients with a PaO_2_/FiO_2_ ratio of less than 150 mmHg [32,34,35]. Although the benefits of proning are well described, the LUNGSAFE study revealed that only 16.3% of patients suffering from ARDS underwent prone positioning [4,36]. Factors associated with low implementation of prone ventilation include: clinician recognition of ARDS, logistical difficulties, fear of complications, and under-recognition of hypoxemia [4,34]. Despite these challenges, efforts should be made to implement daily proning sessions in patients with moderate-to-severe and severe ARDS, until the PaO_2_/FiO_2_ ratio is consistently above 150 mmHg in the supine position [34].

## 7. Neuromuscular Blockade

The utilization of neuromuscular blockade as a ventilator adjunct can potentially benefit patients with ARDS by reducing lung injury caused by patient-ventilator dyssynchrony and strong spontaneous respiratory effort. There is equipoise surrounding the outcome benefit of neuromuscular blockade (NMB) in patients with ARDS [33]. The ACURASYS trial investigated early use of neuromuscular blocking agents in patients with ARDS who were managed with lung protective ventilation. The findings of this study revealed that neuromuscular blockade was associated with improved 90-day mortality and increased ventilator free days [37]. Subsequently, the ROSE trial investigated the use of NMB in patients with moderate-to-severe ARDS. Patients in the intervention group of this trail were managed with an early continuous infusion of cistatracurium, while the control group was managed with lighter sedation targets. Both groups were managed with similar mechanical ventilatory strategies. In contrast to ACURASYS, the ROSE trial revealed no significant endpoint difference between the intervention and control groups at 3, 6, and 12 months. Furthermore, patients in the intervention arm of the ROSE trial were noted to be less physically active and had increased cardiovascular events. A systematic review and meta-analysis of 5 major randomized controlled trials investigating NMB use in ARDS revealed that NMB was associated with reduced risk of barotrauma and improved oxygenation after 48 h, without any worsening of ICU acquired weakness [38]. This conclusion could be related to the finding that NMB was shown to provide benefits in patients of a subgroup requiring more sedation [39].

## 8. Conservative Lung Strategy

Studies have suggested that targeting a conservative strategy can improve gas exchange and improve outcomes in patients with ARDS [40]. In contrast, hypervolemia can increase pulmonary venous pressure and atrial pressure leading to pulmonary edema and a resultant decrease in the PaO_2_/FiO_2_ ratio. In the FACTT trial, patients were divided into two groups, a liberal fluid strategy or conservative strategy. In the conservative strategy group, pulmonary artery occlusion pressure (PAOP) of less than 8 mmHg or central venous pressure (CVP) less than 4 mmHg were used as targets. In the liberal strategy group, the target pressure (PAOP) was 14–18 mmHg and CVP was 10–14 mmHg. Patients managed with the conservative strategy had shortened duration of mechanical ventilation and ICU length of stay [40]. Another large, randomized trial comparing liberal versus conservative strategies of fluid management in patients with acute lung injury also showed better outcomes with conservative fluid management [41]. Patients in the conservative fluid management group had increased ventilator free days, and reduced hospital and ICU length of stay [41]. Another retrospective analysis of patients with ARDS showed that lower pulmonary artery wedge pressure was correlated with better survival [42]. Implementation of a conservative lung strategy in patients with ARDS without contraindications can improve outcomes, reduce the length of ICU and hospital stay, and reduce duration of mechanical ventilation.

## 9. Other Strategies

### 9.1. High Frequency Oscillatory Ventilation

High-frequency oscillatory ventilation (HFOV) uses a constant mean airway pressure (MAP) with pressure variations oscillating at high rates and low tidal volumes to improve ventilation and oxygenation [43]. The MOAT trial was an early study that revealed a non-statistically significant reduction in mortality in patients treated with HFOV, but subsequent trials did not confirm this [44]. The OSCILLATE trial showed that patients treated with HFOV had a higher mortality, and the OSCAR trial revealed no difference in mortality [45,46]. A meta-analysis showed that HFOV can reduce hypoxemia but does not improve survival [47]. Based upon the existing literature, HFOV cannot be recommended as a routine ventilation strategy in patients with ARDS.

### 9.2. Non-Invasive Ventilation

Non-invasive ventilation (NIV) has an important role in the management of hypercapnic respiratory failure, however, its role in the management of acute hypoxic respiratory failure and ARDS is controversial [48,49]. The goal of NIV in ARDS, as well as in other forms of respiratory failure, is to avoid endotracheal intubation and its associated complications. The use of NIV in the management of acute respiratory failure has steadily increased over the years, is associated with improved survival, and results in fewer secondary complications associated with intubation and invasive mechanical ventilation [50]. It is also associated with a decreased mortality in patients with acute on chronic respiratory failure [50]. NIV is often avoided in patients with ARDS citing the association of NIV failure with a worse prognosis [48]. However, it is not clear whether this worse prognosis is related to more severe underlying disease or whether it is a direct result of using NIV and delaying endotracheal intubation [48]. One retrospective observational study revealed that patients who fail NIV have similar outcomes to those intubated early, and those successfully avoiding intubation with NIV use had better outcomes [51]. NIV can be delivered via a face mask or helmet, and the method of delivery can have an impact on outcomes. One single center randomized clinical trial has shown that in patients with ARDS, helmet delivered NIV is associated with lower intubation rates in comparison to face mask delivered NIV [52]. Other reports show that NIV use in ARDS is independently associated with increased ICU mortality, especially in patients with PaO_2_/FiO_2_ ratios of less than 150 [53,54]. The FLORALI trial suggests that while intubation rates of NIV and high-flow oxygen therapy for acute hypoxic respiratory failure resulted in similar intubation rates, high-flow oxygen therapy was superior in regard to 90-day mortality [55].

In patients with COVID-19, the HENIVOT trial showed that helmet NIV (PEEP of 10–12 cm H_2_O and pressure support of 10–12 cm H_2_O) compared to high-flow nasal oxygen therapy showed no significant difference in the number of days free of respiratory support [56]. However, the rate of endotracheal intubation and days free of invasive ventilation at 28 days were significantly lower [56]. Additional research is needed to determine whether, and what method of NIV, should be utilized in the management of patients with ARDS.

### 9.3. Airway Pressure Release Ventilation

Airway pressure release ventilation (APRV) is a mode of ventilation that involves maintaining a continuous high positive pressure for most of the cycle with intermittent release phases, while allowing for spontaneous respiration. APRV may be better tolerated than other modes, allowing for the avoidance of the use of heavy sedation and neuromuscular blockade. Whether APRV should be utilized as an initial ventilatory mode for the management of ARDS is controversial due to lack of high quality evidence [57]. One single center trial has shown that in comparison to classical lung protective ventilation, APRV reduced duration of mechanical ventilation and length of ICU stay when applied early in patients with ARDS [58]. Other low quality evidence exists favoring APRV use in patients at high risk for developing ARDS in comparison to synchronized intermittent mandatory ventilation [59]. Additional research is needed to determine whether and how this mode of ventilation can be best utilized for the management of patients with ARDS.

### 9.4. Venovenous Extracorporeal Membranous Oxygenation (ECMO)

Venovenous (VV) ECMO is believed to function through two main mechanisms: firstly, it improves oxygenation and reduces tissue hypoxia; and secondly, it reduces ventilator induced lung injury by allowing for reduction in the intensity of mechanical ventilation [33]. There have been two major randomized controlled trials that have investigated the use of ECMO in ARDS. The CESAR trial compared patients with severe ARDS who were transferred to an ECMO-capable center or managed with routine care. The primary outcome in the CESAR trial was survival without severe disability at 6 months, which was found to be significantly higher in the ECMO group (67% compared to 43% with conventional management) [60]. The EOLIA trial compared the use of ECMO in severe ARDS to conventional treatment with a crossover to ECMO if required for refractory hypoxemia [61]. The results of this trial revealed a non-statistically significant reduction in mortality in the ECMO group (35% versus 46%), despite a high rate of crossover to ECMO in the control group [33]. A meta-analysis of both studies showed that mortality in patients with dysfunction of 1 or 2 organ systems decreased by almost half with the use of VV ECMO but did not significantly affect mortality in patients who had several failing organ systems [60]. There are important limitations with both studies. Firstly, in the CESAR trial a substantial number of subjects in the ECMO group did not receive ECMO, which complicated the interpretation of final results. Secondly, the EOLIA trial was complicated by crossover to ECMO in 28% of patients receiving conventional treatment, as it was considered unethical to deny ECMO to patients who were persistently hypoxemic despite maximal efforts [61]. This introduced bias into the study and made it difficult to definitively conclude the impact of ECMO on patient outcomes. A systematic review combining the two studies, as well as other reports, showed a statistically significant decrease in mortality with the use of VV ECMO [62]. Indications for VV ECMO include potentially reversible acute severe respiratory failure that is unresponsive to routine ventilation management. ELSO guidelines suggest considering VV ECMO in patients with a mortality risk of at least 50%, and VV ECMO is indicated when the mortality risk is above 80% [63].

VV ECMO is believed to be most beneficial for the management of severe ARDS in younger patients with fewer comorbidities and a reversible etiology of ARDS [33]. Selecting patients for ECMO can be challenging and inclusion criteria vary between institutions. Key factors that are taken into consideration include the severity and etiology of the respiratory failure. Earlier use of ECMO (initial 1–2 days from onset of acute respiratory failure) is associated with improved outcomes [63]. A meta-analysis assessing long term effects of ARDS patients treated with VV ECMO showed that those who received ECMO experienced significantly less psychological morbidity as compared to conventional mechanical ventilation [64]. A recent study also demonstrated that non-ECMO patients had a greater impairment of health-related quality of life [65]. These outcomes remain poorly understood and require further assessment with larger scale prospective studies.

## 10. Conclusions

In this review, we have discussed various ventilatory strategies and ventilatory adjuncts for the management of ARDS. The main objective of the various treatment modalities is to improve outcomes of patients due to the high morbidity and mortality associated with this sinister condition. The most impactful strategies remain lung protective ventilation and prone ventilation. Strategies such as conservative fluid management and neuromuscular blockade are additional adjuvants that can be used in patients with specific clinical situations. Further studies are needed to investigate driving pressure, optimal PEEP, and to determine the role of VV ECMO in the management of ARDS. Evidence-based ventilatory strategies for the management of ARDS have evolved over time and further research is needed to optimize patient management and improve patient outcomes.

## Figures and Tables

**Table 1:** Summary of current guidelines for mechanical ventilation in adult patients with ARDS [6,66].

Society	Recommendation	Strength of Recommendation	Evidence
ATS/ESICM/SCCM	Mechanical ventilation with low tidal volumes and inspiratory pressures	Strong	Moderate
	Daily prone positioning >12 h	Strong	Moderate-high
	Avoid HFOV in patients with moderate or severe ARDS	Strong	Moderate-high
	Mechanical ventilation with higher levels of PEEP for moderate or severe ARDS	Conditional	Moderate
	Recruitment maneuvers should be used	Conditional	Low-moderate
	Additional research needed to recommend use of ECMO in patients with ARDS	Not applicable	Not applicable
FICM/ICS	Mechanical ventilation with low tidal volumes (<6 mL/kg ideal body weight) and plateau pressure (<30 cm H_2_O)	Strong	Moderate
	Daily prone positioning ≥12 h in patients with moderate/severe ARDS	Strong	Moderate
	Avoid HFOV	Strong	Moderate
	Conservative fluid management	Weakly in favor	Low
	Mechanical ventilation with higher levels of PEEP in patients with moderate/severe ARDS	Weakly in favor	Low
	Neuromuscular blocking agents in patients with moderate/severe ARDS	Weakly in favor	Moderate
	Use of ECMO in patients with severe ARDS	Weakly in favor	Very low

Abbreviations: ATS: American Thoracic Society; ESICM: European Society of Intensive Care Medicine; SCCM: Society of Critical Care Medicine Clinical Practice Guideline; FICM: Faculty of Intensive Care Medicine; ICS: Intensive Care Society.
